# Supplementary material for: Factors Affecting the Spread, Diagnosis, and Control of African Swine Fever in the Philippines
Source: Pathogens. 2023 Aug 21;12(8):1068. doi: 10.3390/pathogens12081068 (PMC10459637; doi:10.3390/pathogens12081068)
Supplement: Supplementary file 1 [file pathogens-12-01068-s001.zip › pathogens-2504087-supplementary.pdf]

Article

Factors Affecting the Spread, Diagnosis, and Control of African Swine Fever in the Philippines

Chia-Hui Hsu [1,*], Rachel Schambow [1], Maximino Montenegro [2], Ruth Miclat-Sonaco [3] and Andres Perez [1]

[1] Center for Animal Health and Food Safety, College of Veterinary Medicine, University of Minnesota, Saint Paul, MN 55108, USA; scham083@umn.edu (R.S.); aperez@umn.edu (A.P.)
[2] Pig Improvement Company (PIC) Philippines, Pasig City 1605, Philippines; max.montenegro@genusplc.com
[3] National Livestock Program, Office of the Undersecretary for Livestock, Department of Agriculture, Elliptical Road, Diliman, Quezon City 1101, Philippines; rmiclat@ati.da.gov.ph
* Correspondence: hsu00124@umn.edu

Discussion – Philippines

Two activities will be organized, intended to obtain information from ProgRESSVet participants on (1) knowledge gaps and research needs for ASF in the country, and (2) the value of certain parameters and processes.

Each activity will be organized as a modified "world-café" discussion. Participants will be grouped in tables and a list of questions will be shared with each group. One "rapporteur" will be designated for each table. Results of the discussions will be sequentially shared by each "rapporteur" with the rest of the groups. Conclusions will be listed and different degrees of consensus will be established between and among groups and participants.

Each activity will last approximately 3 hours: introduction and general expectations (10 min), group discussions (45 min), break (10 min), second round of group discussions (45 min), break (10 min), presentations of findings (45 min), conclusions (15 min)

Questions/topics for discussion on each group:

Activity 1: knowledge gaps and research needs for ASF

1. In your opinion and experience, what aspects of the current national ASF control program function well and what remaining challenges exist? What are the things we are doing well and what are the things that could be improved? Which factors do you believe have contributed more to ASF spread in the Philippines? Please provide evidence to substantiate your claim. Think both about the virus, about swine production systems and about human behavior. Why we have not been able to control ASF yet, or what are the things that may help control the disease?

   Allocate your answers to one of each of the following categories:

- Strengths: These are things we are doing well, or that may play in our favor, and that relate to the structure of the swine industry or the disease itself
- Weaknesses: These are things we are not doing so well, or that may play against us, and that relate to the structure of the swine industry or the disease itself
- Opportunities: These are things we are doing well, or that may play in our favor, and that relate to factors other than the structure of the swine industry or the disease itself (e.g. social conditions, economic factors, etc).
- Threat: These are things we are not doing so well, or that may play against us, and that relate to factors other than the structure of the swine industry or the disease itself (e.g. social conditions, economic factors, etc).

2. What are the unknowns? What are the conditions, factors, or situations that we should know to improve our chances of controling the disease?

   Allocated your answers to one of each of the following categories:

- Features of the susceptible population: distribution, number, size, etc.

- Features of the disease: transmission between animals and herds, mechanisms of spread, survivavility in environment, etc.
- Features related to diagnosis, surveillance, and control: accuracy of the diagnostic tests, measures taken by industry to control the disease, etc
- Features related to human behavior: motivations for reporting, incentives for eliminating the disease, structure of the value chain, etc.

Activity 2: Gather information

1. How does ASF get into farms? Imagine that 100 outbreaks are investigated on commercial farms, and the same number of outbreaks is investigated on backyard farms. How many of them, do you estimate, may be attributed to each of the following routes?

	**Commercial Farms**	**Backyard Farms**
Introduction of sick pigs		
Environmental contamination (water courses, rice fields next to the farm, contact with backyard farms, etc)		
Contaminated vehicles entering the farm		
Contaminated people entering the farm		
Feed (feeding with fodders, etc)		
Wild boars or feral pigs		
Rodents, flies, and other potential vectors		
Other		
Total	100	100

2. How does ASF look like?

**Sign**	**Imagine you have 100 Outbreaks, in How Many of them do you Think the Following Signs will be Seen?**	**How many days after introduction of the disease into the farm do you think this sign will first appear?**	**Do you think a producer should be able to notice this sign?**
Increase in mortality or sudden death			
Drop of feed consumption?			
Huddled pigs and other signs of fever			
Reddish in the skin? (Erythema)			
Cyanosis (blue areas) of ears and limbs?			
Abortion			
Constipation followed by diarrhea?			
Diarrhea with fresh blood (Hematochezia)?			
Reluctances to stand up and move (abnormal recumbence)?			
Vomiting			
Vomiting with blood (Hematemesis)			
Pigs bleeding from nose			
Kidney hemorrhages (kidney darker or full of red dots (petechiae))			

Lymphadenomegaly (Enlarged lymph nodes, bigger than normal size)	
Lymph node hemorrhage or necrosis (Darker or full of blood lymph nodes)	
Splenomegaly (Enlarged spleen, bigger than the normal)	
Hydropericardium (Lots of fluid around the heart)	
Hydrothorax (lots of fluid in the toracic cavity)	
Shock lung / Acute Respiratory Distress Syndrome (Red, heavy, wet lungs)	
Pneumonia (Reddish areas in the lungs)	
Hemorrhagic intestinal contents (Blood inside the guts)	

3. What are we doing to control ASF? Imagine that 100 outbreaks are investigated on commercial farms, and the same number of outbreaks is investigated on backyard farms. In how many of them, do you think, these actions will take place?

	**Commercial Farms**	**Backyard Farms**
Disease will be reported to the government		
Animals will be sold as soon as the disease is noticed		
Total depopulation will take place		
Partial (animal-level) depopulation will take place		
Partial (group-level) depopulation will take place		
Nothing is done. Just continue with production		
